# CMR tagging in the polar coordinate system

**DOI:** 10.1186/1532-429X-13-S1-O27

**Published:** 2011-02-02

**Authors:** Abbas N Moghaddam, Yutaka Natsuaki, J Paul Finn

**Affiliations:** 1 UCLA, Los Angeles, CA, USA; 2 Siemens Medical Solution, Los Angeles, CA, USA

## Introduction

Strain of the myocardium is conventionally presented in the polar coordinate system since it adapts best to the morphology of the heart. Strain calculation would be facilitated considerably if the CMR tagging patterns were in the radial or circumferential direction. However, the CMR tagging is implemented mostly in the Cartesian coordinate system as it is prescribed by SPAMM technique in which the gradient fields create only parallel taglines. Radial tagging is not used widely due to SAR problem for tight radial pattern. Implementation of circular tagging, to the best of our knowledge, has not been reported yet. Here we introduce an approach that makes both patterns possible for tagging based on off-resonance excitation. Its theoretical basis and practical details as well as initial results in phantoms and human hearts are presented.

## Methods

A rotating excitation plane in combination with a continuous RF pulse results in substantial excitation on a patterned region using off-resonance effect. A spoiler gradient destroys the net transverse magnetization on the region and generates the tagging with the specified pattern. The actual implementation of these cine sequences was performed on a commercial imaging platform (Siemens Medical Solutions, Erlangen, Germany) by using developmental software (IDEA, VB17; Siemens Medical Solutions).

## Results

**Figure 1:**
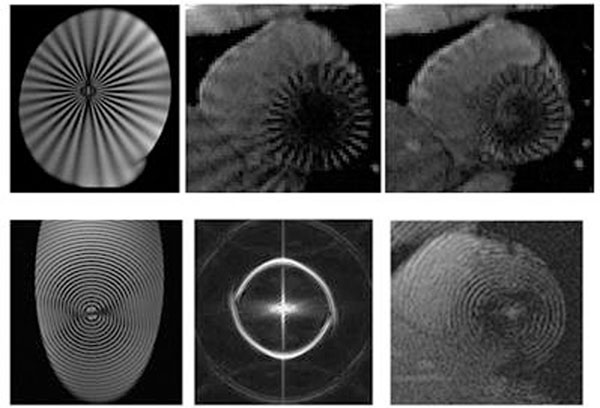
Top: Radial tagging on a stationary uniform phantom in a double oblique imaging plane(Left) and a healthy volunteer at diastole and systole (Middle and Right). The tortional motion of the LV is well pronounced at the posterior wall. Bottom: circular tagging on a phantom (Left), its K-Space representation (Middle), and on a healthy volunteer (Right). The effect of circular tagging is well separated in K-Space.

The developed sequences were tested on phantoms and applied on healthy volunteers (Figure 1). Images acquired by a 1.5T scanner. Other MR parameters are as follows: 250mm FOV, 5mm slice thickness, 250Hz/pixel, 15° flip angle, TE/TR = 4.6/86ms, 128x128 matrix size (256x256 for circular tagging).

## Conclusions

New tagging sequences were theoretically described and actually implemented. The sequences have been successfully tested on phantom and also used to acquire short axis images of the left ventricle. The spatial resolution and density of taglines for the radial tagging are considerably higher compared to its previous schemes and allows for relatively simple derivation of myocardial shear rate and angular strain.

